# Effectiveness of the Portuguese version of Fume in adolescents’ health literacy about tobacco^*^

**DOI:** 10.1590/1518-8345.5455.3513

**Published:** 2022-03-11

**Authors:** Daniela Lourenço Pinto, Heidi Parisod, Johanna Nyman, Tereza Maria Mendes Dinis de Andrade Barroso

**Affiliations:** 1 Escola Superior de Enfermagem de Coimbra, Unidade de Investigação em Ciências da Saúde: Enfermagem, Coimbra, Portugal.; 2 University of Turku, Department of Nursing Science, Finlândia.

**Keywords:** Health Literacy, Mobile Applications, Adolescent, Tobacco, Self Efficacy, Nursing, Letramento em Saúde, Aplicativos Móveis, Adolescente, Tabaco, Autoeficácia, Enfermagem, Alfabetización en Salud, Aplicaciones Móviles, Adolescente, Tabaco, Autoeficacia, Enfermería

## Abstract

**Objective:**

To evaluate the effectiveness of the version translated into European Portuguese of the Fume mobile health game (No Fume), with regard to the health literacy of adolescents in the school context on tobacco-related issues.

**Method:**

A quantitative and quasi-experimental study, with pre-test and post-test evaluation, conducted with a convenience sample of 144 adolescents, divided into an experimental group and a control group, carried out in two public schools serving the 2^nd^ and 3^rd^ cycles of elementary education from a city in the district of Coimbra, central region of Portugal.

**Results:**

Among the adolescents who used No Fume (experimental group), a statistically significant and favorable evolution was verified in the negative expectations about smoking (*p* = 0.033).

**Conclusion:**

No Fume revealed a positive effect on the negative expectations about smoking among the adolescents. Investments should be made in developing No Fume in order to maximize its potential for use since, as an educational technology based on gamification, it may come to contribute to the expansion and dissemination of innovative interventions that promote adolescents’ mental health.

## Resumo

**Objetivo:**

Avaliar a efetividade da versão traduzida para português europeu do *mobile health game Fume (No Fume)*, no que diz respeito à literacia em saúde de adolescentes em contexto escolar sobre as questões relacionadas com o tabaco.

**Método:**

Estudo quantitativo, quase-experimental, com avaliação pré-teste e pós-teste, com uma amostra por conveniência de 144 adolescentes, dividida por um grupo experimental e um grupo de controle, realizado em duas escolas públicas do 2º e 3º ciclo do ensino básico de uma cidade do distrito de Coimbra, região centro de Portugal.

**Resultados:**

Verificou-se, nos adolescentes que utilizaram o *No Fume* (grupo experimental), uma evolução favorável, estatisticamente significativa, nas expetativas negativas sobre fumar (*p* = 0,033).

**Conclusão:**

O *No Fume* revelou um efeito positivo nas expetativas negativas sobre fumar dos adolescentes. Deve investir-se no desenvolvimento do *No Fume* para maximizar o seu potencial de utilização pois, enquanto tecnologia educativa baseada na *gamificação*, poderá contribuir para o alargamento e disseminação de intervenções inovadoras promotoras da saúde mental dos adolescentes

## Abstract (es)

**Resumen:**

**Objectivo**

Evaluar la efectividad de la versión traducida al portugués europeo del *mobile health game Fume (No Fume)* en la alfabetización en salud de los adolescentes en un contexto escolar sobre temas relacionados con el tabaco.

**Método**

Estudio cuantitativo, cuasiexperimental, con evaluación pretest y postest, con una muestra por conveniencia de 144 adolescentes dividida en un grupo experimental y un grupo de control, realizado en dos escuelas públicas del segundo y tercer ciclo de educación básica de una ciudad en el distrito de Coímbra, región central de Portugal.

**Resultados**

Los adolescentes que utilizaron No Fume (grupo experimental), tuvieron una evolución favorable estadísticamente significativa en las expectativas negativas sobre fumar (*p* = 0,033).

**Conclusión**

*No Fume* reveló un efecto positivo sobre las expectativas negativas sobre fumar en los adolescentes. Se debe invertir en el desarrollo de *No Fume* para maximizar su potencial de uso porque, como tecnología educativa basada en la *gamificación*, puede contribuir a la expansión y difusión de intervenciones innovadoras que promuevan la salud mental de los adolescentes.

**Highlights**

(1) No Fume is the Portuguese version of the mobile health game Fume. (2) No Fume’s effect on health literacy on tobacco among adolescents was evaluated. (3) No Fume revealed a positive effect in negative expectations about smoking. (4) In order to maximize its potential for use, No Fume is being further developed.

## Introduction

At a world level, tobacco consumption is a serious public health problem, accounting for millions of deaths every year and a large burden of disease, assessed in millions of disability-adjusted life years worldwide^1^.

The situation is also, and particularly, alarming in the younger population, insofar as, and despite the mean age of consumption initiation in Portugal is pointed out as 16 years old^2^, it is known that, at age 13, around 11.7% of the adolescents have already smoked cigarettes and that the majority consider the occurrence of sporadic consumption to be of little or no risk^3^.

Thus, it is essential to develop health literacy in adolescence, defined as the set of “cognitive and social skills and the person’s ability to access, understand and use information in order to promote and maintain good health”^4^. Adolescence is a key period for developing health literacy, as it is at this stage that, in addition to the process of identity construction, the foundations for a healthy lifestyle are also consolidated^5^.

Worldwide, the use of serious games has been recurrent in several age groups and contexts, with positive results in terms of, for example, knowledge acquisition, development of affective and social skills and change in behavior^6^. In the health area, serious games have been used both in promotion and prevention^7^ and in health recovery, showing positive effects, namely with regard to the children’s and adolescents’ health^8^ ^,^ ^9^. Serious games consist of video games developed with goals beyond entertainment, their primary functions being learning and behavioral change^10^. A number of studies have shown the effect of serious games, namely, in improving the adolescents’ knowledge related to alcohol, tobacco and other drugs^9^ and in the favorable evolution of other determinants of tobacco-related health literacy^11^.

In Finland, at the University of Turku, precisely, a mobile health game aimed at children and adolescents was developed, with the objective of improving health literacy related to tobacco issues and preventing its consumption at this stage of development^12^. The effect of this game was rated against a website with contents similar to that of the game, although not playable and, in addition to the acceptability of the game by the adolescents being better than in relation to the website, in the short term, favorable changes were verified with regard to the attitudes towards consumption of cigarettes and the positive and negative expectations in relation to tobacco^13^.

Considering adolescence as a developmental stage of priority intervention in the issues related to tobacco consumption, the significant adherence by this target population to the new technologies and the positive effects of mobile health games in adolescents’ health literacy, the translation of Fume^12^ to European Portuguese was considered relevant, as well as the evaluation of its effectiveness, since there was no other mobile health game for the same purpose. Thus, this study was carried out, whose objective was to evaluate the effectiveness of the version of Fume translated into European Portuguese (designated as *No Fume*), with regard to the determinants of health literacy on the issues related to tobacco among adolescents in the school context.

## Method

### Type

This is a quantitative and quasi-experimental study, with a Control Group (CG) and an Experimental Group (EG), with pre-test and post-test evaluation.

### Locus

The study was carried out in two public schools serving the 2^nd^ and 3^rd^ cycles of elementary education, from the same parish council, which belong to a city in the district of Coimbra, central region of Portugal.

### Period

Data collection took place in April and May 2019.

### Population

In its original version, the Fume mobile health game was developed by Parisod, et al. for children and adolescents aged between 10 and 13 years old, in order to reach them before a possible first experience with tobacco^12^. Likewise, its translated version into European Portuguese, *No Fume*, has adolescents belonging to the same age group as its target population.

### Selection criteria

Two public schools from the same parish council from a city in the district of Coimbra, central region of Portugal, were selected. The EG belonged to one of the participating schools and the CG to the other school selected. Five 6^th^ grade classes participated in each of the groups, with an equivalent number of students per class. All the students who submitted their consent were included in the sample.

Regarding the Portuguese version, to be eligible to participate in the study to assess its effectiveness, it was defined that, as inclusion criteria, children and adolescents had to be between 10 and 13 years old and understand and be able to communicate in Portuguese.

### Sample definition

The sampling method used was non-probabilistic, of the convenience type.

### Study variables

The translated version of the Fume mobile health game, which is designated as No Fume, constituted the independent variable.

By addressing several determinants of health literacy, the Fume mobile health game aims at supporting the motivation and ability to access, understand and use information related to tobacco and promote a life free from tobacco consumption in early adolescence, seeking to meet the teenagers’ expectations^12^. Fume integrates a quiz, consisting of questions on the knowledge about tobacco, and four other minigames, using sound and visual effects to highlight the positive effects of a life without tobacco consumption and the negative consequences of its use^12^.

The other four minigames included in the game are as follows: *Bus minigame*, where the player chooses between different characters and tries to take the bus with the selected character, depending on the character chosen and the player’s performance, the game presents different results that seek to show the effects of tobacco in sports and in health; *environment minigame*, in which the player has to clean the tobacco garbage that exists in the environment as quickly as possible, the objective of this game being to evidence the effects of tobacco in the environment; *piggy bank minigame*, the player has to protect the piggy bank of character *Mr. Nicotine Dependency*, preventing that person from being hit by a hammer, intending, in this way, to demonstrate nicotine dependence and the financial consequences of tobacco consumption; and the *accepting and refusing tobacco minigame*, where the players choose how to act in a situation where they are offered tobacco products, the objective of this game being to encourage resistance to peer pressure and to say no to tobacco.

**Figure 1:**
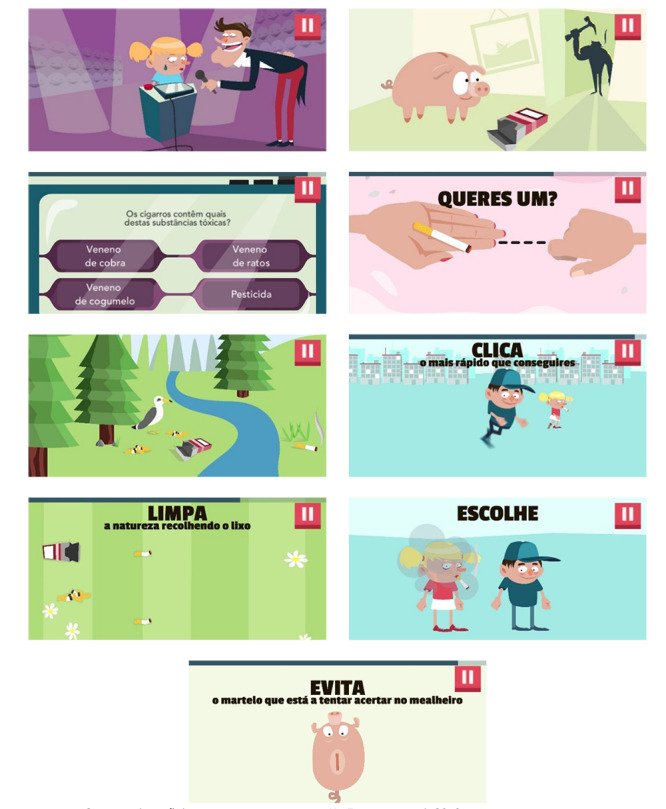
Screenshots of the minigames found in No Fume. Portugal, 2019

No Fume maintained its general structure similar to the original version (quiz and minigames), with only minor changes being made to adapt it to the cultural context. Figure 1

Regarding the determinants of tobacco-specific health literacy, all minigames are geared towards attitudes, beliefs/perceptions and experiences, as the effects of tobacco are shown through the use of a knowledge-based approach that appeals to the emotions to provide experiences, and influence attitudes and beliefs. All games also include interpretation of health messages, as they all integrate tobacco-related verbal and non-verbal messages that adolescents may face. With the exception of the accepting or rejecting tobacco game, the quiz and all the mini-games are geared towards knowledge related to tobacco. The expectations regarding the roles are addressed in the environment game, through preventive tasks that the adolescents can practice in their communities; self-efficacy is addressed in the bus game and in the accepting or rejecting tobacco game, as these include tasks related to tobacco and everyday life whose difficulty increases as the game progresses. These tasks also allow the players to track what happens to the characters according to their tobacco-related choices, support positive feelings and offer incentives and scores that allow for comparisons between players. Motivation and self-efficacy are addressed both in the bus game and in the accepting or rejecting tobacco game; and the expectations regarding responsibilities and social skills are addressed, respectively, in the environment game and in the accepting or rejecting tobacco game^12^.

Therefore, the determinants of health literacy about tobacco were considered as dependent variables: attitudes towards tobacco use; reasons for tobacco use; motivation to refuse tobacco in the future; knowledge about tobacco; positive and negative expectations about tobacco; and anti-tobacco self-efficacy.

The attitudes towards tobacco use concern the way each person feels about the substance and their opinion about it^13^. The reasons for using tobacco are related to how its use is understood by each person in terms of the social message it conveys^14^. With regard to the positive and negative expectations about tobacco, these are related to the perceptions that each individual has about the substance and the type of arguments developed for and against^13^. Expectations can be positive, in the sense that the individual can consider that tobacco has positive consequences, or negative, if the person considers it as a substance with negative consequences.

Self-efficacy was also considered as a dependent variable, which consists of the beliefs that each person has about their own abilities to perform a certain action, whether to perform a task or make a choice^13^.

### Instruments used to collect information

The attitudes towards tobacco use were assessed using the Attitudes Towards Tobacco Use item, resulting from the translation and adaptation of the scale with the same name^11^. The reasons for tobacco use were assessed using the Tobacco-Use Motives, translated from the instrument of the same name^11^. In relation to the motivation to refuse tobacco use in the future, this was assessed through the Motivation to Decline Tobacco Use in the Future item, which resulted from the translation into European Portuguese of the scale with the same name^11^. 

Knowledge about tobacco was assessed through the open-ended question “What do you know about the consequences of smoking cigarettes?”, with its answers being analyzed by dichotomizing the variable into “knows at least one consequence” or “does not know any consequence”.

The positive expectations variable was evaluated using the Positive Expectations about Smoking Scale and the negative expectations variable using the Negative Expectations about Smoking Scale, which were translated and adapted to European Portuguese from the original POS-SOES (Positive Smoking Outcome Expectation Scale) and NEG-SOES (Negative Smoking Outcome Expectation Scale)^15^ scales, respectively.

In this study, self-efficacy was assessed using the Anti-Smoking Self-Efficacy Scale, translated and adapted to European Portuguese from the original instrument (ASSES)^15^. Permissions to use the scales in this study were received from the copyright holders.

### Data collection

Data collection took place during school activities, at times defined with the class director, in order to disturb the dynamics as little as possible. The researcher responsible for the study and three junior researchers, trained for the purpose, were involved in the data collection procedures.

Initially (pre-test), each student completed a general data questionnaire and translated versions of the data collection instruments (30 minutes).

The adolescents were asked, on the day of the intervention, to bring their smartphone/tablet with due authorization of the guardians, and there are also mobile devices with the game installed, in case they were necessary. Thus, the intervention was then implemented to the EG by the researchers (45 minutes, divided into 20 minutes for game download and preparation of the intervention, 20 minutes for the intervention with the game and 5 minutes for conclusion of the intervention). Therefore, the intervention consisted of a guided game session of No Fume for 20 minutes and the recommendation to play it in the following 2 weeks, according to their interest. After these 2 weeks (post-test), the same instruments were applied to both groups, at the same time points, as in the pre-test, with the exception of the general data questionnaire and with introduction of a questionnaire on the acceptability of No Fume for the EG (20 minutes).

### Data treatment and analysis

All the data collected were organized in a database and analyzed in the IBM SPSS Statistics software, version 25.0. To assess the differences between the groups, in the pre-test, with regard to the sociodemographic variables, Mann-Whitney’s *U* test was used, as well as the Chi-Square and Fisher›s Exact tests. To characterize the sample, descriptive analysis was used, through the analysis of absolute and percentage frequencies. Analysis of the mean was also used, as a measure of central tendency, as well as the analysis of the standard deviation, as a measure of dispersion. For the analysis of the data regarding the intragroup differences and differences between groups, in the pre-test and post-test, statistical analysis was used and, having verified that the sample did not follow normal distribution, it was decided to use non-parametric tests. To assess the differences between the groups for the variables under study, in the pre-test and in the post-test, the Mann-Whitney *U test was used*. To analyze the evolution of each group (intragroup) between the pre-test and the post-test moments, the Wilcoxon test was used, in order to compare the variables under study within each of the groups between two different moments. *p*p <0.05 was considered as statistically significant.

In the context of the studied variables, a favorable evolution is understood as one that promotes healthy behaviors. In this sense, in the case of the determinants: attitudes towards tobacco use; reasons for tobacco use; motivation to refuse tobacco use in the future; and positive expectations about smoking, a favorable evolution is related to a decrease. In the case of the determinants of knowledge about tobacco, negative expectations about smoking and self-efficacy, a favorable evolution translates into an increase.

### Ethical aspects

To carry out this study, all the recommended ethical procedures for the type of research developed were followed, and this study was submitted to the Ethics Committee of the Health Sciences Research Unit: Nursing (*Unidade de Investigação em Ciências da Saúde: Enfermagem*, UICISA: E) From the Nursing School of Coimbra and a favorable opinion was obtained (P521-09/2018). Formal authorization was also requested from the entities where the study took place, which was authorized in writing, via e-mail, by the Directors of the two groups of participating schools.

If they agreed to participate, signed informed consent was required, both by the child or adolescent and their guardian, who ensured that authorization to participate was voluntary and that there would be no penalty in case of non-participation, as well as that the data would be confidential and anonymous, and used only for the exclusive use of this study, with the questionnaires being coded to allow assessment before and after participation in the intervention and data pairing, without identifying the participants.

## Results

Regarding the sample of this study, it was constituted by an EG and by a CG. The results were evaluated by applying the aforementioned self-administered instruments.

IConsidering the consents obtained at the two schools, the CG included 87 adolescents and the EG, 57, with a mean age of 11.43 years old (SD = 0.587). In relation to the comparison between the CG and the EG, there was homogeneity in the variables studied, with the exception of the “nationality” variable, with the participants’ characterization according to the group presented in Table 1.

**Table 1:** Participants’ characterization according to the group. Portugal, 2019

Variable		Control Group (n=87)	Experimental Group (n=57)	*p* ^*^
**Gender**	Female	52 (59,8%)	32 (56,1%)	0,666^†^
	Male	35 (40,2%)	25 (43,9%)	
**Age**	10 years old	2 (2,3%)	0 (0%)	0,407^++^
	11 years old	51 (58,6%)	32 (56,1%)	
	12 years old	32 (36,8%)	22 (38,6%)	
	13 years old	2 (2,3%	2 (5,3%)	
**Nationality**	Portuguese	85 (97,8%)	47 (82,4%)	0,002^§^
	Foreign	2 (2,2%)	10 (17,6%)	
**Allowance**	Yes	25 (28,7%)	19 (33,3%)	0,431^†^
	No/No answer	73 (83,9%)	47 (82,4%)	
***Smartphone***	Yes	73 (83,9%)	47 (82,4%)	0,750^†^
	No/No answer	14 (16,1%)	10 (17,6%)	

^*^ *p* = Significance level (*p* < 0,05); ^†^ The Chi-Square (X^2^) test was used; ^++^ *U* de Mann-Whitney test was used; ^§^ Fisher’s Exact test was used.

To assess the differences between the groups in relation to the variables under study, at the pre-test and post-test moments, the non-parametric Mann-Whitney’s U test was used Table 2.

In relation to the attitudes towards tobacco use, reasons for tobacco use, motivation to refuse tobacco use in the future and positive expectations about smoking, there were no statistically significant differences between the CG and the EG, not even at the pre- test or post-test moments. However, it should be noted that the CG started from a more favorable position with regard to these variables.

With regard to the negative expectations about smoking, while in the pre-test the differences between the CG and the EG were not statistically significant (*p* = 0.332), there were differences with statistical significance (*U* = 2035.500; *p* = 0.031) in the post-test. There is a favorable evolution in the EG, suggesting effectiveness of the intervention with *No Fume* in this variable.

In relation to anti-tobacco self-efficacy, in the pre-test, the GC starts from a more favorable position than the EG. There were statistically significant differences between the groups, both in the pre-test (*p* = 0.018) and in the post-test (*p* = 0.020), although the evolution is favorable in both groups. 

Regarding knowledge about tobacco, all adolescents in the EG were able to identify at least one consequence of tobacco, both in the pre-test and in the post-test. In relation to the CG, it was verified that, both in the pre-test and in the post-test, one adolescent did not answer the question, and the others were able to identify at least one consequence of tobacco.

**Table 2:** Intergroup assessment using Mann-Whitney’s *U* test. Portugal, 2019

Variable of Interest	Pre-test						Post-test					
	Control Group (*n* = 87)		Experimental Grup (*n* = 57)		*U* ^§^	*p* ^||^	Control Group (*n* = 87)		Experimental Grup (*n* = 57)		*U* ^§^	*p* ^||^
	*M* ^*^(*SD* ^†^)	MR^‡^	*M* ^*^(*SD* ^†^)	MR^‡^			*M* ^*^(*SD* ^†^)	MR^‡^	*M* ^*^(*SD* ^†^)	MR^‡^		
Attitudes towards Tobacco Use	1,25 (0,533)	70,22	1,39 (0,701)	75,97	2281,500	0,275	1,20 (0,478)	69,25	1,42 (0,801)	77,46	2196,500	0,098
Reasons for Tobacco Use	3,45 (1,097)	68,09	3,65 (1,308)	76,89	2096,000	0,100	3,44 (1,178)	69,32	3,70 (1,336)	77,36	2202,500	0,128
Motivation to Refuse Tobacco Use in the Future	1,22 (0,562)	69,08	1,30 (0,499)	76,40	2200,000	0,148	1,23 (0,522)	69,45	1,29 (0,497)	74,74	2214,500	0,304
Positive Expectations about Smoking (Scale)	4,40 (1,689)	67,00	4,79 (1,578)	79,54	2021,000	0,066	4,34 (1,938)	67,16	4,58 (1,569)	79,31	2034,500	0,070
Negative Expectations about Smoking (Scale)	10,79 (1,639)	69,46	11,02 (1,768)	75,83	2232,500	0,332	10,57 (2,631)	67,40	11,40 (1,557)	80,29	2035,500	0,031
Anti-Tobacco Self-Efficacy (Scale)	54,52 (7,187)	76,48	52,84 (6,548)	60,11	1766,000	0,018	56,26 (6,392)	77,93	54,74 (5,921)	61,91	1876,000	0,020

*M = Mean; ^†^SD = Standard Deviation; ^‡^MR = Mean Rank; ^§^U = Mann-Whitney test; ^||^p = Significance level (p <0.05).

To assess the evolution in each group between the two assessment moments (pre-test and post-test) in relation to the variables under study, the non-parametric Wilcoxon test was used (Table 3).

Regarding the determinants: attitudes towards tobacco use, reasons for tobacco use, motivation to refuse tobacco use in the future and positive expectations about smoking, the differences between the pre-test and the post-test were not significant in either of the groups.

Regarding the negative expectations about smoking, the differences were significant in the EG (p = 0.033), which suggests effectiveness of the intervention with No Fume, as shown in Table 3, with a higher proportion of adolescents in the EG, which presents a favorable evolution in relation to the CG.

As for the anti-tobacco self-efficacy variable, there were significant differences in both groups between the pre-test and post-test moments (CG: p = 0.006; EG: p = 0.010); however, there was a higher proportion of adolescents who presented a favorable evolution in the EG

**Table 3:** Intragroup assessment using the Wilcoxon Test. Portugal, 2019

	**Control Group (CG) (*n* = 87)**					**Experimental Group (EG) (*n* = 57)**				
Variable of Interest	*Status*	*n* ^*^	MR^†^	*Z* ^‡^	*p* ^§^	*Status*	*n* ^*^	MR^†^	*Z* ^‡^	*p* ^§^
Attitudes towards Tobacco Use^||^	Better	8	6,75	-1,291	0,197	Better	9	9,28	-0,092	0,927
	Worse	4	6,00			Worse	9	9,72		
	No change	75				No change	39			
Reasons for Tobacco Use^||^	Better	9	9,11	-0,272	0,786	Better	11	8,27	-0,165	0,869
	Worse	8	8,88			Worse	8	12,38		
	No change	70				No change	36			
Motivation to Refuse Tobacco Use in the Future^||^	Better	7	7,50	0,000	1,000	Better	4	5,00	-0,333	0,739
	Worse	7	7,50			Worse	5	5,00		
	No change	72				No change	46			
Positive Expectations about Smoking (Scale)^||^	Better	19	17,45	-0,275	0,783	Better	27	17,67	-1,257	0,209
	Worse	16	18,66			Worse	12	25,25		
	No change	50				No change	18			
Negative Expectations about Smoking (Scale)^¶^	Better	26	19,77	-0,499	0,617	Better	19	11,76	-2,134	0,033
	Worse	17	25,41			Worse	5	15,30		
	No change	43				No change	33			
Anti-Tobacco Self-Efficacy (Scale)^¶^	Better	35	28,34	-2,766	0,006	Better	33	23,50	-2,582	0,010
	Worse	17	22,71			Worse	13	23,50		
	No change	30				No change	9			

* *n* = Number of adolescents; ^†^MR = *Mean Rank*; ^‡^ *Z* = Wilcoxon Test; ^§^ *p* = Significance level (*p* < 0,05); ^||^A favorable evolution corresponds to a decrease in the score; ^¶^favorable evolution corresponds to an increase in the score.

## Discussion

As already mentioned, *No Fume* is the first mobile health game related to health literacy associated with tobacco issues in Portugal, specifically aimed at the stage of early adolescence, for the particular age group from 10 to 13 years old, and with the specific objective of promoting a life without tobacco, that is, to prevent consumption of this substance

With regard to the effectiveness of No Fume, no statistically significant effects were found in most of the variables under study, with the exception of negative expectations about smoking, which could be an indicator of some weaknesses of the intervention. This could be related to the content of the game, too short period of time the game was played or lack of debriefing session.

In relation to the attitudes towards tobacco use, in general, most of the adolescents reveal a negative attitude towards smoking cigarettes and both groups presented negative attitudes at both evaluation moments, verifying that the use of No Fume had no statistically significant effects for this determinant of health literacy about tobacco. However, in the study of the original version^14^, Fume, the author verified a statistically significant effect of using this version on the attitudes towards smoking cigarettes.

Analyzing this aspect is important because the fact that an individual presents a negative attitude towards certain behavior, for example, is a protective factor for not adopting it, as a person’s behavior tends to be consistent with their attitudes. At this level, the theory of planned behavior makes important contributions to the explanation of human social behavior, considering that attitudes towards certain behavior can be used, with a high degree of precision, to predict behavioral intentions, considering that these intentions, in combination with the perception of control of the behavior, may explain the variations that occur^16^.

Although the differences were not statistically significant in the case of No Fume in relation to the attitudes towards tobacco, and of all the minigames included in No Fume are directed to this determinant^12^, both in the CG and in the EG it was verified that, in the post-test, more adolescents indicated the “very stupid” option when compared to the pre-test moment. This means that, even without the intervention, the attitudes towards tobacco changed, which may be related, on the one hand, to the simple fact of approaching the theme and, on the other, to completing the scales, which can lead to the adolescents having the subject matter more present, meditating on their own beliefs about tobacco, reflecting on this at the post-test moment. 

In relation to the reasons for tobacco use, for this determinant, effectiveness of the intervention with No Fume was not verified. These results are in line with those verified with Fume^14^. However, as a result of data analysis, it was verified that, in both groups, the adolescents presented, at the pre-test moment, very favorable rates with regard to the reasons for tobacco use, that is, indicators of the option for healthy behaviors. Social reasons are one of the factors recognized in the literature as one of the main influences for the adoption of risk behaviors among young people, such as tobacco or alcohol consumption^17^; therefore, health promotion interventions in general, and tobacco consumption prevention in particular, should focus on empowering young people with social skills that allow them to interpret and deal with the frequent social pressures that arise in this life cycle period.

The analysis of the motivation to refuse tobacco use in the future revealed that there were no statistically significant effects resulting from the intervention with No Fume and, for this determinant of health literacy about tobacco, no significant differences were found with Fume either^14^. Despite this, in a study developed in this area^13^, this was a valued determinant of health literacy specific to the tobacco, with the adolescents considering that, in the case of a teenager who has personal or sports-related goals, for example, these could be a motivation to refuse tobacco use.

This reinforces the importance of young people setting goals and targets, since some studies consider this an important factor, for example, as an indicator of resilience^18^. Importance is also attributed to the establishment of goals since, as define goals, individuals end up monitoring their behavior more than usual^19^ and, therefore, they are more alert to the consequences and influence that other behaviors may have in achieving their goals.

With regard to the intervention with No Fume, eventually, it may also be necessary to also study the several factors that can lead an adolescent to refuse to consume tobacco, in order to optimize the game accordingly and, thus, include more minigames or questions that address these factors, so that more adolescents see themselves in them.

In relation to knowledge about tobacco, all adolescents in the EG were able to identify at least one consequence of tobacco, both in the pre-test and in the post-test. The results remained between the two evaluation moments in the case of the EG, since all were able to identify at least one consequence of smoking cigarettes at both moments, which is of significant importance to highlight, as it reveals that adolescents already have knowledge about the harmful effects of this substance. Thus, it is considered that interventions aimed at adolescents in this context should not only aim at improving knowledge about tobacco, but should also take into account the various other components of tobacco-related health literacy, which may present so much or more importance than the adolescent’s knowledge about the substance and its consequences.

In relation to the expectations about tobacco, with regard to the positive expectations about smoking, there were no statistically significant effects of the intervention with No Fume. However, a clinical gain is verified in the EG, as most of the adolescents in this group presented a favorable evolution, while in the CG the majority did not present changes. In the study of Fume^14^, it was possible to verify the existence of significant differences in the group that used the game, between the pre-test and post-test moments.

Due to the relevance of the positive expectations about smoking in the behavior under analysis, it is important to emphasize that stress relief is one of the factors identified by young people as an aspect they consider positive in tobacco, and this expectation is a factor that assumes significant importance with regard to susceptibility to the onset of tobacco consumption^20^. In this sense, while being a positive expectation related to tobacco consumption, stress relief should be an element to be integrated in future games of this nature, considering that the most common false positive beliefs about tobacco should be sought and these should be further explored and demystified when intervening with the adolescents.

Regarding the negative expectations about smoking, effectiveness of the intervention with *No smoke* was verified, these results being consistent with the study of Fume^14^. Analysis of this determinant of health literacy about tobacco is an important aspect insofar as expectations determine behavior^19^. A study that sought to analyze the relationship between the perception of health risks of tobacco consumption and smoking behavior in a sample of young adults verified that the individuals who considered that tobacco consumption was pleasant, that it helped to deal with problems or with stress, that it allowed them to relax or was something they could do when they were bored, ran the risk of being smokers and, on the other hand, those who considered the existence of health risks and negative consequences were less likely to become smokers^21^.

With regard to anti-tobacco self-efficacy, the results did not show effectiveness of the intervention with No Fume. Although both groups, experimental and control, have evolved in the same direction, the favorable evolution is proportionally higher in the adolescents subjected to the intervention. In the same sense, effectiveness of the intervention with Fume^14^ ^,^ ^19^; therefore, at a stage where the central task is the construction of identity, it seems essential that the health promotion strategies focus on this construct.

The nurse, who knows adolescence as a transition process, plays an essential role in the analysis of all the determinants of tobacco-specific health literacy and in the development of intervention programs aimed at preventing tobacco consumption and, thus, promoting adolescents’ mental health.

With regard to acceptability of No Fume, most of the adolescents considered it to be “cool” or “very cool”, which is in line with the results of the study of Fume^11^.

With regard to the limitations, it is worth mentioning the following: the study design of this research, since quasi-experimental studies present as a weakness the fact that the individuals are not randomly distributed; the disproportionality of the sample (CG: 87; EG: 57); however, through the comparability study, it was verified that the groups were equivalent, with the exception of nationality; and the very short intensity of the intervention, as it consisted only of a guided session with the game. In relation to this last limitation, the model used in Finland was reproduced with Fume, and it was verified that the adolescents had some doubts about the theme of tobacco and that the 20-minute session with the game was insufficient. The need to carry out a debriefing after the game session was felt; therefore, in the future, planning of this intervention may come to include time for such purpose.

Although the results obtained do not reflect effectiveness of the intervention with No Fume on the determinants of health literacy: attitudes towards tobacco use, reasons for tobacco use, motivation to refuse tobacco use in the future, positive expectations about smoking and anti-tobacco self-efficacy, it would be important to survey the games that considered these variables and to analyze how they were addressed and what their results are with regard to health promotion among adolescents, in order to improve No Fume in the future, as well as for the development of future Nursing interventions in this domain.

## Conclusion

The intervention based on the No Fume mobile health game only showed favorable effects on the adolescents’ negative expectations about smoking, which is why it needs to be revised and updated, particularly in its minigames, in order to respond to the objective proposed and to improve health literacy related to tobacco among the adolescents. 

As a gamification-based educational technology, No Fume may come to contribute to the expansion and dissemination of interventions that will promote health literacy in areas as specific as this one, related to the health behaviors. 

Nurses, particularly those specialized in mental health, should be involved in the development of innovative technologies to promote the adolescents’ mental health. In addition to allowing a greater number of adolescents to be reached at the same time, the use of mobile health games has the particularity of having the ability to draw interest and, thus, combine a recreational component with an educational component.

So that No Fume may come to be used as an integrated strategy in an intervention for the prevention of tobacco use aimed at adolescents aged between 10 to 13 years old, this needs to be improved in order to maximize its use potential. Further development of the Fume game intervention has been started already
